# The prevalence and predictors of intimate partner violence among pregnant women attending a midwife and obstetrics unit in the Western Cape

**DOI:** 10.1017/gmh.2018.9

**Published:** 2018-05-08

**Authors:** M. Malan, M.F. Spedding, K. Sorsdahl

**Affiliations:** Alan J. Flisher Centre for Public Mental Health, Department of Psychiatry and Mental Health, University of Cape Town, Western Cape, South Africa

**Keywords:** Domestic violence, interpersonal violence, intimate partner violence, pregnancy, postnatal depression

## Abstract

**Background.:**

Intimate partner violence (IPV) during pregnancy is prevalent across the world, but more so in low- and middle-income countries. It is associated with various adverse outcomes for mothers and infants. This study sought to determine the prevalence and predictors of IPV among pregnant women attending one midwife and obstetrics unit (MOU) in the Western Cape, South Africa.

**Methods.:**

A convenience sample of 150 pregnant women was recruited to participate in the study. Data were collected using several self-report measures concerning the history of childhood trauma, exposure to community violence, depression and alcohol use. Multivariable logistic models were developed, the first model was based on whether any IPV occurred, the remaining models investigated for physical-, sexual- and emotional abuse.

**Results.:**

Lifetime and 12-month prevalence rates for any IPV were 44%. The 12-month IPV rates were 32% for emotional and controlling behaviours, 29% physical and 20% sexual abuse. The adjusted model predicting physical IPV found women who were at risk for depression were more likely to experience physical IPV [odds ratios (ORs) 4.42, 95% confidence intervals (CIs) 1.88–10.41], and the model predicting sexual IPV found that women who reported experiencing community violence were more likely to report 12-month sexual IPV (OR 3.85, CI 1.14–13.08).

**Conclusion.:**

This is the first study, which illustrates high prevalence rates of IPV among pregnant woman at Mitchells Plain MOU. A significant association was found between 12-month IPV and unintended pregnancy. Further prospective studies in different centres are needed to address generalisability and the effect of IPV on maternal and child outcomes.

## Background

Violence is a complex behavioural phenomenon which takes on many forms across a variety of contexts. Violence can have detrimental effects for those who encounter it, often resulting in bodily harm; mental, physical and emotional suffering; loss of productivity; and fatality, representing an increased burden for social and public health sectors (DeVries *et al.*, 2014; Wuest *et al.*, 2015). South Africa is a country with a long history of violence, arguably rooted in systemic racism and apartheid legislation, exerted by a minority authoritarian party against the disempowered, within political, social, judicial, informal, public and domestic settings (Moffette, 2006). Though we have entered a post-apartheid period, violence is still seen to penetrate all sectors of our society and is very common.

While literature from low- and middle-income countries (LMIC) focuses on violence against women in the general population (Hamberger & Larsen, 2015; Katiti *et al.*, 2016), pregnancy has been identified as a period of increased risk for exposure to intimate partner violence (IPV) (WHO, 2011; Bernstein, *et al.*, 2016). IPV is a well-documented phenomenon of behaviour in the context of human relationships (WHO, 2005; Sarkar, 2008; Silva *et al.*, 2011). IPV is defined as ‘patterns of emotional, sexual and/or physical violence and economic intimidation by an intimate partner in conditions of coercive power’ (Patel *et al.*, 2011, 1). IPV during pregnancy is of concern as the possible consequences pose risk to both mother and the unborn child (Taillieu & Brownridge, 2010; Mobasheri *et al.*, 2013). IPV during pregnancy has been associated with various negative outcomes, including pregnancy-related deaths, inadequate ante-natal care, labour complications, emotional distress, depression, anxiety and harmful neonatal consequences such as low birth weight (Moreno *et al.*, 2005; Charles & Perreira, 2007; Howard *et al.*, 2013).

In South Africa, statistics among pregnant woman reveal that IPV rates range between 27 and 48% (DeVries *et al.*, 2014). For example, a cross-sectional community-based descriptive study (Hoque *et al.*, 2009), found that the prevalence rates of IPV for the past month, among 340 rural pregnant women in KwaZulu Natal, were at 31%. Another study investigating prevalence rates of IPV among pregnant woman from antenatal clinics in the metropolitan of Soweto found that more than 50% of 1395 pregnant women interviewed, reported experiencing IPV in the previous 12 months (Dunkle *et al.*, 2004). In addition, analyses of a subsample of 544 mothers from socially disadvantaged peri-urban communities outside of Cape Town indicated that they had experienced IPV within the past year (Koen *et al.*, 2016).

IPV is not an isolated occurrence, but part of an interaction between social, personal, emotional and psychological factors. To develop appropriate strategies for IPV prevention among pregnant women, an understanding of the associated risk and protective factors is essential. Several socio-demographic factors have been found to be associated with IPV. Women from low socio-economic backgrounds have been found at higher risk for experiencing IPV compared with a woman from higher socio-economic backgrounds (Patel *et al.*, 2011; Capaldi *et al.*, 2012). Supporting this statement, a National survey in England established factors such as low household income, deprived education and low social class to be associated with lifetime prevalence for IPV (Khalifeh *et al.*, 2013). Contradictory to this, a systematic review from Africa revealed no difference between women whom were employed compared with those who were not (Shamu *et al.*, 2011). Other socio-demographic variables that have been found to be associated with IPV include the level of education and age. In support, one cross-sectional study of 1502 pregnant women attending healthcare facilities in Mpumalanga, South Africa, found that more than 55% of those women had less than a grade 12-level education and that the dominant age was between 18 and 24 years (Matseke *et al.*, 2012).

A commonly noted risk factor associated with IPV is the harmful use of alcohol during pregnancy (WHO, 2010; Labato *et al.*, 2011). Alcohol use has been associated with countless personal harms including violence and aggression; elevated rates of IPV; general familial friction as well as poor parenting styles, as it affects both cognitive and physical functioning, often reducing the ability of self-control and increasing the likeliness of violent altercations (WHO, 2006; Stanley, 2008). Alcohol consumption is noted to be high among South Africans; especially in the Western Cape where rates of alcohol abuse exceed 20% (Herman *et al.*, 2009; Pasche & Myers, 2012). Alcohol use has been associated with increased risk of perpetrating acts of violence, increased risk for being a victim of IPV; increased risk for exposure to IPV as well as increased risk of resorting to alcohol as a means of coping with IPV (WHO, 2010; Simonelli *et al.*, 2014).

One of the social factors commonly identified as a risk factor for IPV is being exposed to or witnessing violence as a child (Norman *et al.*, 2010; Moore *et al.*, 2011; Capaldi *et al.,* 2012; Shamu *et al.*, 2016). A notable association between childhood abuse and IPV has been identified, demonstrating that women who have experienced abuse during their childhoods are more likely to experience IPV during their adult years, compared with women who had not experienced abuse during childhood (Whitfield *et al.*, 2003; Widom *et al.*, 2008; Patel *et al.*, 2011). Dunkle *et al.* (2004) found that childhood sexual violence was established as a major risk factor for experiencing IPV in adulthood. Outcomes of childhood abuse have predominantly centred on victims re-experiencing abuse in their adulthood, either by becoming perpetrators of violence themselves or by becoming victims of violence again (Omduff *et al.*, 2001; Widom *et al.*, 2008).

Violence should not be viewed as an isolated event, but rather one that is manifested and fuelled by greater influences, one of which is the community in which one resides (Hamberger *et al.*, 2015). Mitigating environmental factors are also considered as part of the causal factors associated with violence (Chen *et al.*, 2016). Frustrations associated with low levels of socio-economic opportunities and high levels of unemployment may be implicated in high levels of crime and violence, as well as high rates of alcohol and substance abuse sometimes found in poorer communities (Raghavan *et al.*, 2006; Beyer *et al.*, 2015). Low-income communities especially face problems with gangsterism and violent crimes. It has been reported that high rates of exposure to community violence often results in the notion that violence is acceptable (Abrahams *et al.*, 2005).

Not surprisingly, mental health problems have been shown to be highly correlated with experiences of IPV. These include psychological suffering and emotional distress; as well as mental health conditions, including depression, anxiety and post-traumatic stress disorder (Fortin *et al.*, 2011; Umubyeyi *et al.*, 2014). Recently, Woollett & Hatcher (2016) found that in South Africa, symptoms of depression increase among individuals who experienced elevated rates of exposure to violence in both domestic and community settings, supporting the argument that violence has an impact on the mental functioning of an individual (Iverson *et al.*, 2015). Pregnant women are possibly additionally vulnerable as evidenced by the high prevalence rates of mental health problems that occur during this time (Holden *et al.*, 2012). It is important to note that abuse during pregnancy highly correlates with symptoms of depression, as mental health outcomes have been conceptualised as a consequence of IPV during the peri- and post-partum periods of pregnancy (Rochat *et al.*, 2011, Jackson *et al.*, 2015).

To date, there have been no studies investigating the prevalence of IPV in a midwife and obstetrics unit (MOU) in the Western Cape, South Africa, and the associations between alcohol use, depression, childhood abuse and exposure to community violence. This study was conducted at Mitchell's Plain MOU, a primary level maternity facility offering pregnant women antenatal care throughout the pregnancy, as well as HIV testing and counselling. The facility also has a labour ward for deliveries as well as post-natal care. Understanding the frequency of IPV within this community is essential to identify possible need for psychosocial interventions. The present study attempts to address this gap by answering the following two-pronged research question: What is the prevalence of IPV among women attending one antenatal clinic in the Western Cape, South Africa and what are the risk factors associated with IPV among this population.

## Methods

### Setting

Data were collected at an MOU in the Western Cape, South Africa. This community forms part of one of the largest townships in the Western Cape, South Africa. Townships are commonly used to describe urban or peri-urban communities that are largely economically and geographically underdeveloped.

### Participants

A convenience sample of 150 pregnant women attending antenatal care appointments at the MOU was asked to participate in this study. Participants had to be 18 years or older, pregnant (any term during pregnancy), a registered patient at the MOU, as well as being willing and able to participate in an interview in either English or Afrikaans.

### Study procedure

Recruitment of participants occurred over a 2-month period (November–December 2015). Potential participants were approached while they waited for the clinic. Appointments for interviews were scheduled for those who interest in participation on days and times that were most convenient for the participants. Sampling took place 5 days of the week for ~5 h/day.

G*Power version 3.1.9 software (Faul *et al.*, 2009) was used to calculate the sample size for a multivariate logistic regression analysis. The alpha was set at 0.05 and desired power at 0.80. The estimated sample size is 175 participants. Unfortunately, due to time and budgetary constraints only 150 women participated.

Prior to the interview, each participant was again informed about the voluntary nature of the study, as well as the conditions of confidentiality and anonymity concerning their involvement, and then asked to complete the consent form. Interviews of approximately 45 min in duration were conducted by the first author, a qualified Registered Counsellor, in a private office. Questionnaires were made available in both English and Afrikaans and were read aloud to each participant. Participants each received a voucher of ZAR50 (approximately US$3.60) for a local grocery store as compensation for their time. Participants who required further intervention after the interview, where assessed and given referrals to social workers, counsellors or mental health nurses, as deemed necessary by the researcher (a registered trauma counsellor).

### Measures

Data were collected using the following measures:

#### Socio-demographics questionnaire

Information was collected regarding participant's race, age, level of education, current employment status and marital status, whether the pregnancy was planned or unplanned, the number of previous pregnancies and pregnancy gestation.

#### WHO interpersonal violence questionnaire (IPVQ) (WHO, 2000)

This is a survey questionnaire used to screen for past and current experiences of IPV (*lifetime and 12 months*). It includes subscales for the assessment of emotional abuse and controlling behaviour as well as physical and sexual abuse. Questions such as ‘Have you ever been hit, slapped, kicked or otherwise physically hurt by your current or previous intimate partner?’ are included. While it has not yet been validated for the use in South Africa, local studies have shown good reliability rates (Koen *et al.*, 2016).

#### Childhood trauma questionnaire (CTQ) (Bernstein *et al.*, 1994)

This is a 28-item self-report inventory that provides brief, reliable and valid screening for childhood histories of abuse and neglect. This instrument follows a five-factor format, enquiring about five different types of maltreatment – emotional, physical and sexual abuse, and emotional and physical neglect. It has been validated in both clinical and community settings and has been translated into several languages (Patel *et al.*, 2011). Studies have found an internal consistency coefficient close to 0.80, indicating good reliability for responses over a period of time (Hernandez *et al.*, 2013; Grassi-Oliveria *et al.*, 2014).

#### Exposure to community violence questionnaire (WHO, 2000)

This measure was adapted from the Children's Exposure to Community Violence Questionnaire (Richters & Martinez, 1990). The frequency of lifetime exposure to violence (through visual and auditory senses) is measured by this tool. It determines the incidence of violence in the home and in the general neighbourhood. This includes questions such as: ‘Have you seen someone being beaten up?’ and ‘Have you seen a gun in your home?’ This measure has not been validated in clinical settings (Beyer *et al.*, 2015).

#### The Edinburgh postnatal depression scale (EPDS) (Cox et al., 1987)

This is one of the most widely used instruments to screen for both ante- and postnatal depressive symptoms. This is a self-report rating scale with an internal consistency score of 0.83 (Bunevicius *et al.*, 2009). It is low and middle- income countries (Akena *et al.*, 2012) and includes questions such as ‘I have been so unhappy that I have been crying’. Multiple validation studies have found the EPDS to be a reliable screening instrument (Shrestha *et al.*, 2016).

#### Alcohol use disorder identification test (AUDIT) (WHO, 2001)

This measure screens for hazardous and harmful patterns of alcohol consumption. It includes questions such as: ‘How often do you have a drink containing alcohol?’ and ‘How often during the last year have you found that you were not able to stop drinking once you started?’ A systematic review of the psychometric properties of the AUDIT, found great performance rates for the tool, across a variety of settings for the 47 studies analysed (Shrestha *et al.*, 2016). Studies have found it to be a reliable and effective tool to detect harmful alcohol consumption behaviours (Pitpitan *et al.*, 2013; Sabri *et al.*, 2014).

### Data analysis

The Statistical Package for Social Science (SPSS) (23.0) was used to analyse the data. Frequency distributions and descriptive statistics were calculated for categorical and continuous variables. The unadjusted associations between IPV as the dependent variable, and participant demographic characteristics, history of childhood abuse, self-report alcohol abuse and perception of community violence as independent variables, were analysed. In addition, multivariate logistic models were developed to control for demographics and socio-economic variables (including gender, age, race and marital status), alcohol abuse, community violence and childhood trauma. The first was based on whether any IPV occurred (lifetime and 12 months), while the remaining three models investigated IPV for physical abuse, sexual abuse and emotional abuse for both lifetime and 12 months experience. The results of the regression models are reported as odds ratios (ORs) with 95% confidence intervals (CIs).

## Results

The socio-demographic details of participants are detailed in Table 1. A total of 150 women were recruited to participate in the study. Of these women, the majority were unemployed (*n* = 93, 62%) and between the ages of 18 and 30 years old (*n* = 103, 68.7%). Just over half of the respondents completed high school (*n* = 79, 52.7%). Respondents reported that many of them indicated that their current pregnancy was unplanned (*n* = 115, 76.7%). Notably of the 115 participants who indicated that their pregnancies were unplanned only 32 (65.3%) specified being in an intimate relationship. More than half of the participants, (*n* = 83, 55.3%) reported that they were in their second trimester of pregnancy (weeks 13–28). Respondents reported high levels of childhood trauma (*n* = 123, 82%) and witnessing community violence (*n* = 94, 62.7). It was found that 38.7 and 25.3% met criteria for depression and alcohol, respectively.

**Table 1.** Socio-demographic, pregnancy-related and psychosocial characteristics of sample

	Total (*N* = 150)	Single (*N* = 101)	In an intimate relationship (*N* = 49)	
	*N* (%)	*N* (%)	*N* (%)	
Age				
18–30	103 (68.7)	74 (73.3)	29 (59.2)	0.08
31 and older	47 (31.3)	27 (26.7)	20 (40.8)	
Race				
Black	67 (44.7)	50 (49.5)	17 (34.7)	0.07
Coloured	81 (54.0)	49 (48.5)	32 (65.3)	
White	2 (1.3)	2 (2.0)	0 (0.0)	
Employment status				
Employed	57 (38.0)	36 (35.6)	21 (42.9)	0.31
Unemployed	93 (62.0)	65 (64.4)	28 (57.1)	
Education				
Did not complete high school	71 (47.3)	50 (49.5)	21 (42.9)	0.44
Completed high school	79 (52.7)	51 (50.5)	28 (57.1)	
Pregnancy planned				
Planned pregnancy	35 (23.3)	18 (17.8)	17 (34.7)	0.02
Unplanned pregnancy	115 (76.7)	83 (82.2)	32 (65.3)	
Gravidity (m, s.d.)	2.5 (1.6)	2.3 (1.4)	3 (1.7)	0.01
Parity (m, s.d.)	1.3 (1.4)	1.1 (1.3)	1.7 (1.5)	0.11
Miscarriage				
Yes	34 (22.7)	20 (19.8)	14 (28.6)	0.23
No	116 (77.3)	81 (80.2)	35 (71.4)	
Pregnancy gestation				
Weeks 3–12	48 (32.0)	29 (28.7)	19 (38.8)	0.20
Weeks 13–28	83 (55.3)	61 (60.4)	22 (44.9)	
Weeks 29–40 and more	19 (12.7)	11 (10.9)	8 (16.3)	
Mental health				
High-risk depression	58 (38.7)	44 (43.6)	14 (28.6)	0.07
Low-risk depression	91 (61.3)	57 (56.4)	35 (71.4)	
Alcohol use				
Low-risk drinker	112 (74.7)	68 (67.3)	44 (89.8)	0.07
High-risk drinker	38 (25.3)	33 (32.7)	5 (10.2)	
Childhood trauma				
No childhood trauma	27 (18.0)	17 (16.8)	10 (20.4)	0.11
Yes, childhood trauma	123 (82.0)	84 (83.2)	39 (79.6)	
Community violence				
Low community violence	56 (37.3)	33 (34.7)	28 (57.1)	0.66
High community violence	94 (62.7)	66 (65.3)	21 (42.9)	

Both the 12-month and lifetime prevalence rates for IPV were calculated to be 44.7%. Physical violence was the most common form of abuse experienced in the women's lifetime (*n* = 70, 46.7%), followed by emotional abuse and controlling behaviours (*n* = 66, 44%) and sexual violence (*n* = 24.7%). Most types of IPV victimisation were reported to occur during the second trimester of pregnancy (*n* = 39, 58.2%) (see Table 2). It was noted that nearly 80% of the 150 women interviewed, reported that they had never (in their lifetime) opened a police case for assault. No significant differences were noted for prevalence rates between single woman and woman in intimate partner relationships (*p* > 0.05).

**Table 2.** Any IPV in past 12 months and associated variables

	% Yes (*N* = 67)	% No (*N* = 83)	Unadjusted OR (95% CI)	Adjusted OR (95% CI)
Age				
18–30	52 (77.6)	51 (61.4)	1.00	1.00
31 and older	15 (22.4)	32 (38.6)	0.46 (0.22–0.95)	0.41 (0.14–1.19)
Race				
Black	28 (41.8)	39 (47.0)	1.00	1.00
Coloured	39 (58.2)	44 (53.0)	1.29 (0.68–2.48)	1.46 (0.64–3.34)
Employment status				
Employed	20 (29.9)	37 (44.6)	1.00	1.00
Unemployed	47 (70.1)	46 (55.4)	1.90 (0.96–3.73)	1.24 (0.53–2.90)
Education				
Did not complete high school	31 (46.3)	40 (48.2)	1.00	1.00
Completed high school	36 (53.7)	43 (51.8)	1.09 (0.57–2.06)	1.50 (0.65–3.46)
Pregnancy intended				
Planned pregnancy	8 (11.9)	27 (32.5)	1.00	1.00
Unintended pregnancy	59 (88.1)	56 (67.5)	3.56 (1.50–8.48)	3.36 (1.21–9.38)
Miscarriage				
Yes	15 (22.4)	19 (22.9)	1.00	1.00
No	52 (77.6)	64 (77.1)	1.03 (0.48–2.22)	0.33 (0.08–1.41)
Pregnancy gestation				
Weeks 3–12	17 (25.4)	31 (37.3)	1.00	1.00
Weeks 13–28	39 (58.2)	44 (53.0)	1.61 (0.78–3.36)	1.90 (0.78–4.62)
Weeks 29–40 and more	11 (16.4)	8 (9.6)	2.51 (0.85–7.43)	2.70 (0.71–10.24)
Mental health				
Low depression	27 (40.3)	65 (78.3)	1.00	1.00
High Depression	40 (59.7)	18 (21.7)	5.35 (2.62–10.93)	6.56 (2.70–15.97)
Alcohol use				
Low-risk drinker	47 (70.1)	65 (78.3)	1.00	1.00
High-risk drinker	20 (29.9)	18 (21.7)	1.54 (0.73–3.22)	0.70 (0.28–1.76)
Childhood trauma				
No childhood trauma	14 (20.9)	13 (15.7)	1.00	1.00
Yes, childhood trauma	40 (59.7)	70 (84.3)	0.70 (0.31–1.62)	1.30 (0.46–3.71)
Community violence				
Low community violence	50 (74.6)	44 (53.0)	1.00	1.00
High community violence	17 (25.4)	39 (47.0)	2.61 (1.30–5.25)	1.45 (0.61–3.43)

The unadjusted and adjusted associations between participant characteristics and the experience of emotional and controlling behaviours in the past 12 months are reported in Table 3. After adjusting for the effects of other variables, women who are emotionally abused are more likely to experience symptoms of depression (OR 6.42, CI 2.51–16.41) and identified themselves as Coloured. Investigations into the unadjusted and adjusted associations between participant characteristics and the experience of physical IPV in the past 12 months are reported in Table 4. After adjusting for the effects of other variables, women who were at risk for depression were more likely to experience physical IPV (OR 4.42, CI 1.88–10.41) than women not at risk for depression.

**Table 3.** Emotional and controlling behaviours IPV in the past 12 months

	% Yes (*N* = 48)	% No (*N* = 48)	Unadjusted OR (95% CI)	Adjusted OR (95% CI)
Age				
18–30	37 (77.1)	66 (64.7)	1.00	1.00
31 and older	11 (22.9)	36 (35.3)	0.55 (0.25–1.20)	0.31 (0.09–1.07)
Race				
Black	14 (29.2)	53 (52.0)	1.00	1.00
Coloured	34 (70.8)	47 (64.1)	2.74 (1.31–5.72)	3.79 (1.448–9.75)
Employment status				
Employed	13 (27.1)	44 (43.1)	1.00	1.00
Unemployed	35 (72.9)	58 (56.9)	2.04 (0.97–4.31)	1.23 (0.48–3.12)
Education				
Did not complete high school	23 (47.9)	48 (47.1)	1.00	1.00
Completed high school	25 (52.1)	54 (52.9)	0.97 (0.49–1.92)	1.64 (0.67–4.04)
Pregnancy intended				
Planned pregnancy	8 (16.7)	27 (26.5)	1.00	1.00
Unintended pregnancy	40 (83.3)	75 (73.5)	1.80 (0.75–4.33)	1.16 (0.40–3.34)
Gravidity (m, s.d.)	2.5 (1.6)	2.6 (1.6)	0.84 (0.46–1.54)	0.16 (0.13–1.40)
Parity (m, s.d.)	1.3 (1.6)	1.3 (1.4)	1.22 (0.64–2.33)	0.14 (0.74–9.28)
Miscarriage				
Yes	12 (25.0)	22 (21.6)	1.00	1.00
No	36 (75.0)	80 (78.4)	0.83 (0.37–1.85)	0.36 (0.08–1.59)
Pregnancy gestation				
Weeks 3–12	11 (22.9)	37 (36.3)	1.00	1.00
Weeks 13–28	27 (56.3)	56 (54.9)	1.62 (0.72–3.66)	1.56 (0.59–4.14)
Weeks 29–40 and more	10 (20.8)	9 (8.8)	3.74 (1.21–11.50)	3.48 (0.89–13.67)
Mental health				
Low depression	17 (35.4)	75 (73.5)	1.00	1.00
High depression	31 (64.6)	27 (26.5)	5.07 (2.42–10.59)	6.42 (2.51–16.41)
Alcohol use				
Low-risk drinker	35 (72.9)	77 (75.5)	1.00	1.00
High-risk drinker	13 (27.1)	25 (24.5)	1.14 (0.52–2.50)	0.49 (0.18–1.34)
Childhood trauma				
No childhood trauma	13 (27.1)	14 (13.7)	1.00	1.00
Yes, childhood trauma	35 (72.9)	88 (86.3)	0.35 (0.57–3.52)	0.65 (0.23–1.86)
Community violence				
Low community violence	8 (16.7)	48 (47.1)	1.00	1.00
High community violence	40 (83.3)	54 (52.9)	0.39 (0.96–1.12)	2.48 (0.91–6.77)

**Table 4.** Physical IPV in past 12 months

	% Yes (*N* = 44)	% No (*N* = 106)	Unadjusted OR (95% CI)	Adjusted OR (95% CI)
Age				
18–30	18 (40.9)	69 (65.1)	1.00	1.00
31 and older	26 (59.1)	37 (34.9)	0.55 (0.24–1.23)	0.53 (0.17–1.60)
Race				
Black	34 (77.3)	49 (46.2)	1.00	1.00
Coloured	10 (22.7)	55 (51.9)	1.29 (0.63–2.63)	1.44 (0.62–3.35)
Employment status				
Employed	13 (29.5)	44 (41.5)	1.00	1.00
Unemployed	39 (88.6)	62 (58.5)	1.69 (0.80–3.60)	1.04 (0.43–2.52)
Education				
Did not complete high school	23 (52.3)	48 (45.3)	1.00	1.00
Completed high school	21 (47.7)	58 (54.7)	0.76 (0.37–1.53)	0.82 (0.35–1.93)
Pregnancy intended				
Planned pregnancy	5 (11.4)	30 (28.3)	1.00	1.00
Unintended pregnancy	39 (88.6)	76 (71.7)	3.08 (1.11–8.56)	2.70 (0.87–8.39)
Gravidity (m, s.d.)	2.4 (1.4)	2.6 (1.6)	0.90 (0.70–1.14)	0.08 (0.11–1.14)
Parity (m, s.d.)	1.2 (1.3)	1.3 (1.5)	0.92 (0.71–1.20)	0.81 (0.87–9.97)
Miscarriage				
Yes	11 (25.0)	23 (21.7)	1.00	1.00
No	33 (47.7)	83 (78.3)	0.83 (0.37–1.90)	0.23 (0.05–1.01)
Pregnancy gestation				
Weeks 3–12	13 (29.5)	35 (33.0)	1.00	1.00
Weeks 13–28	25 (56.8)	58 (54.7)	1.16 (0.53–2.56)	0.89 (0.36–2.22)
Weeks 29–40 and more	29 (13.6)	13 (12.3)	1.24 (0.39–3.96)	0.78 (0.20–3.01)
Mental health				
Low depression	16(36.4)	76 (71.7)	1.00	1.00
High depression	28 (63.6)	30 (28.3)	4.43 (2.10–9.34)	4.42 (1.88–10.41)
Alcohol use				
Low-risk drinker	28 (63.6)	84 (79.2)	1.00	1.00
High-risk drinker	16 (36.4)	22 (20.8)	2.18 (1.01–4.73)	1.42 (0.59–3.46)
Childhood trauma				
No childhood trauma	10 (22.7)	17 (16.0)	1.00	1.00
Yes, childhood trauma	34 (77.3)	89 (84.0)	0.02 (0.98–1.05)	0.98 (0.35–2.78)
Community violence				
Low community violence	12 (27.3)	44 (41.5)	1.00	1.00
High community violence	32 (72.2)	62 (58.5)	0.03 (0.96–1.12)	1.02 (0.41–2.56)

The unadjusted and adjusted effects of participant characteristics on the experience of any 12-month IPV are displayed in Table 4. After adjusting for the effects of the other variables in the model, being depressed (OR 6.56, 95% CI 2.70–15.97), and reporting that this pregnancy was unplanned (OR 3.36, 95% CI 1.21–9.38) was significantly associated with the reporting any IPV in the past 12 months.

The unadjusted and adjusted associations between participant characteristics and the experience of sexual IPV in the past 12 months (by an intimate partner) are reported in Table 5. After adjusting for the effects of other variables, women reported experiencing community violence were more likely to report 12-month sexual IPV (OR 4.42, CI 1.88–10.41), than women who reported no exposure to community.

**Table 5.** Sexual IPV in the past 12 months

	% Yes (*N* = 30)	% No (*N* = 120)	Unadjusted OR (95% CI)	Adjusted OR (95% CI)
Age				
18–30	24 (80.0)	79 (65.8)	1.00	1.00
31 and older	6 (20.0)	41 (34.2)	0.48 (0.18–1.27)	0.57 (0.14–2.28)
Race				
Black	13 (43.3)	54 (45.0)	1.00	1.00
Coloured	17 (56.7)	64 (53.3)	1.17 (0.50–2.75)	1.15 (0.43–3.07)
Employment status				
Employed	9 (30.0)	48 (40.0)	1.00	1.00
Unemployed	21 (70.0)	72 (60.0)	1.33 (0.53–3.32)	0.97 (0.35–2.71)
Education				
Did not complete high school	13 (43.3)	58 (48.3)	1.00	1.00
Completed high school	17 (56.7)	62 (51.7)	1.45 (0.62–3.53)	1.30 (0.47–3.61)
Pregnancy intended				
Planned pregnancy	5 (16.7)	30 (25.0)	1.00	1.00
Unintended pregnancy	25 (83.3)	90 (75.0)	1.50 (0.50–4.48)	1.23 (0.38–4.02)
Gravidity (m, s.d.)	2.1 (1.2)	2.6 (1.6)	0.71 (0.31–1.62)	0.13 (0.09–1.36)
Parity (m, s.d.)	1.0 (1.4)	1.4 (1.4)	1.14 (0.47–2.76)	0.20 (0.63–8.43)
Miscarriage				
Yes	8 (26.7)	26 (21.7)	1.00	1.00
No	22 (73.3)	94 (78.3)	0.71 (0.27–1.87)	0.30 (0.06–1.58)
Pregnancy gestation				
Weeks 3–12	7 (23.3)	41 (34.2)	1.00	1.00
Weeks 13–28	22 (73.3)	61 (50.8)	2.11 (0.83–5.40)	2.08 (0.72–6.01)
Weeks 29–40 and more	1 (3.3)	18 (15.0)	0.33 (0.04–2.84)	0.23 (0.02–2.30)
Mental health				
Low depression	13 (43.3)	79 (65.8)	1.00	1.00
High depression	17 (56.7)	41 (34.2)	2.21 (0.93–5.28)	2.15 (0.82–5.67)
Alcohol use				
Low-risk drinker	20 (66.7)	92 (76.7)	1.00	1.00
High-risk drinker	10 (33.3)	28 (23.3)	1.30 (0.52–3.28)	1.09 (0.38–3.13)
Childhood trauma				
No childhood trauma	10 (33.3)	17 (14.2)	1.00	1.00
Yes, childhood trauma	20 (66.7)	103 (85.8)	0.01 (0.97–1.05)	0.40 (0.14–1.20)
Community violence				
Low community violence	4 (13.3)	52 (43.3)	1.00	1.00
High community violence	26 (86.7)	68 (43.3)	0.87 (1.00–1.18)	3.85 (1.14–13.08)

## Discussion

Several important findings were made. First, the prevalence of IPV among pregnant woman attending an MOU in the Western Cape, South Africa was high. Second, the study identified four main variables as risk factors for IPV in this sample of pregnant women. Significant associations were found to exist between IPV and depressive symptoms; IPV and unintended pregnancies; as well as IPV and exposure to community violence. Furthermore, 82% of all respondents reported experiencing high levels of childhood trauma; 62% had witnessed community violence (*n* = 94, 62%), while possible cases of depression and alcohol dependence problems were detected in 38 and 25%, respectively.

Similar to other studies (Graham-Kevan & Archer, 2011; Connor-Smith *et al.,* 2011), the most common form of abuse experienced by women in this sample was physical violence (46%), then emotional abuse and controlling behaviours (44%), followed by sexual violence (24%). Recent data from a systematic review showed that between 4 and 29% of pregnant women from LMIC will experience some form of IPV (Mobasheri *et al.*, 2013). This study's prevalence rates are closely aligned to systematic reviews that have investigated the prevalence of IPV in Africa generally and South Africa specifically, found that up to 40% of pregnant women have experienced some form of IPV in their lifetime (Shamu *et al.*, 2011).

Of the four predictors of IPV identified in this study, age was the only socio-demographic variable found to be significantly associated with IPV. Women who were younger (18–30 years old) were more likely to experience IPV than women who were older than 30. Burgos-Soto *et al.* (2014) have argued that older women may experience more helplessness and detachment from their circumstances as a result of many years of abuse, making them less likely to disclose possible abuse. Unlike other studies (e.g., Brownridge *et al.*, 2011; Capaldi *et al.*, 2012), no association was found between IPV and other socio-demographic variables such as level of education, relationship status or employment status.

Experiencing depressive symptoms during pregnancy was also found to predict elevated IPV in this sample. The results showed that women with symptoms of depression were four times more likely to experience physical IPV than those who were asymptomatic. Increased symptoms of depression were also found to be significantly associated with any form of IPV in the previous 12 months. Depression may also be an outcome/consequence of IPV rather than a predictor; however as this study was cross-sectional, no directional relationship could be determined. These findings are consistent with studies that have found mental health problems to be highly correlated with experiences of IPV (Rao *et al.*, 2012; Iverson *et al.*, 2015; Jackson *et al.*, 2015). In particular, IPV during pregnancy has been shown to be significantly associated with depressive symptoms (Adams *et al.*, 2013; Howard *et al.*, 2013). Notably, other studies conducted in Cape Town have highlighted a much stronger association between IPV and depressive symptoms. For example, Hartley *et al.* (2011) found that women in their sample who experienced abuse in their relationships in the previous 12 months were eight times more likely to report depressive symptoms.

The association between IPV and depression appears to highlight the impact that violence has on the mental health functioning of the individuals exposed to it. However, while no directional link between depressive symptoms and IPV can be inferred from this data, some research has shown that individuals with mental health problems, such as major depressive disorder, are at risk for becoming victims of abuse or ill-treatment (Elbogen & Johnson, 2009; Howard *et al.*, 2013). One systematic review and meta-analysis of longitudinal studies investigating the association between IPV and depression found that IPV was associated with incident depressive symptoms and depressive symptoms with incident IPV, among women (Devries *et al.*, 2014). It is not clear why depressive symptoms might cause IPV; however, Devries *et al.* (2014) posit that these symptoms may lead to poor choices in a partner.

Unintended pregnancy was found to be another predictor of IPV in this sample. Women who reported experiencing emotional and controlling behaviours in the previous 12 months were three times more likely to have an unintended pregnancy than women who reported experiencing no abuse. Remarkably, more than 70% of the study's respondents indicated that their current pregnancy was unintended. Furthermore, of those who reported any form of IPV in the past 12 months, 88% of the women in this sample indicated that their current pregnancy was unintended. Studies investigating the association between IPV and unintended pregnancy are limited. However, it does appear to be a relatively widely recognised risk factor (Pallitto *et al.*, 2005; Mantell *et al.*, 2009). One study in Brazil found that women who experienced violence before becoming pregnant were more than 1.5 times more likely to have unintended pregnancies (Azevedo *et al.*, 2013). Given the limited research, it is not clear what the nature of the association is between unintended pregnancies and IPV. It is possible that women with violent partners experience intimidation, control or coercion from their partners regarding their reproductive choices (Pallitto *et al.*, 2005; Azevedo *et al.,* 2013).

The fourth predictor of IPV identified in this study was exposure to community violence. Data from this study showed that women who reported witnessing community violence were four times more likely to report 12-month sexual IPV, than women who reported no exposure to community violence. Very little research has been conducted to investigate the relationship between IPV and exposure to community violence. However, recent data have shown that the Western Cape has significantly high levels of violence within its communities (Prinsloo *et al.*, 2016), while Cape Town was ranked 9^th^ on the list of the 50 most violent communities in the world (Leggit, 2004; Weatstone, 2016). It is possible that this finding simply reflects the levels of emotional abuse and controlling behaviours that members of this community are exposed to, which extends to IPV.

Where South African policy is concerned, there has been very little recognition of IPV as a major health concern, nor has the development of strategies to effectively deal with IPV been attended to. Currently, the National Mental Health Policy Framework for South Africa (Freeman *et al.*, 2013) makes no reference to IPV. This is concerning given the high levels of mental health problems found among victims of IPV, including depression, post-traumatic stress disorder and anxiety (Hartley *et al.*, 2011; Lagdon *et al.*, 2014; van Heyningen *et al.*, 2016). Given the high prevalence of IPV among pregnant woman, as well as the range of problematic outcomes associated with it, developing a policy that deals specifically with IPV is a matter of urgency. These policies might include the training of professional caregivers to detect IPV victims during routine care, in addition to providing sensitive and appropriate care, intervention and support.

The study had several limitations. First, the study's sample size is small and drawn from one site, making any generalisation to even this setting as well as other parts of South Africa or elsewhere impossible. The substantial prevalence rate found is supported by other studies in the Cape Town area (Abrahams *et al.*, 2013) as well as other extremely poor areas in South Africa (Dunkle *et al.*, 2004). However, given that this MOU is one of the largest in the Western Cape, South Africa (Western Cape Gov., 2016) it does offer some insight into the level of violence experienced by a pregnant woman from this specific population and setting. Second, the researcher is only fluent in English and Afrikaans and so was only able to administer the measures in those languages. Feasibility issues compromised the use of the Xhosa versions of the study's measures, which were additionally limited by the reliance on self-report methods. In addition, basic statistical analytical strategies were employed, limiting the ability to determine the interactions/effects of other possible mediators of IPV.

Recommendations for future research might include longitudinal designs investigating the experience of IPV postnatally and whether there are differences before and after the birth. Longitudinal studies might also provide important data about the long-term impact that IPV has on the mother and child. This would help to inform the development of appropriate intervention strategies and programmes for those affected. The effectiveness of screening and intervention programmes should be investigated. Further research could help examine and identify ways to incorporate screening interventions for IPV among pregnant woman, in primary health care settings. This data this could aid in detecting IPV and linking woman to resources such as non-profit organisations, social services and support groups or legal services.

## Conclusion

This is the first study of IPV to be conducted with pregnant women receiving antenatal care at the Mitchells Plain MOU. These findings are consistent with findings from other SA studies. The results from this study identified three major risk factors associated with IPV during pregnancy; high levels of unintended pregnancies, witnessing community violence and experiencing depressive symptoms. They also highlight the urgency for the development of appropriate policy and strategies to address IPV, particularly among pregnant women. Due to the high levels of IPV among pregnant woman, healthcare institutions and practitioners need to find new ways to identify, contain and provide adequate intervention and support for the victims.
